# Lifestyle Drugs: Concept and Impact on Society

**DOI:** 10.4103/0250-474X.73902

**Published:** 2010

**Authors:** S. Z. Rahman, V. Gupta, Anupama Sukhlecha, Y. Khunte

**Affiliations:** Department of Pharmacology, Jawaharlal Nehru Medical College, AMU, Aligarh-202 002, India; 1 Department of Pharmacology, Maulana Azad Medical College, New Delhi - 110 002, India; 2 Department of Pharmacology, MP Shah Medical College, Jamnagar - 361 008, India

**Keywords:** Lifestyle drugs, lifestyle medicine, socioeconomic impact

## Abstract

Lifestyle has changed from being an indicator of the overall well being of an individual to a cause of disease and now “lifestyle” has itself become an object of medical attention. Alcohol has been used enormously as one of the oldest ‘lifestyle’ drugs, and currently sildenafil citrate (Viagra), the drug of choice for erectile dysfunction, exemplifies a turning point in the era of modern lifestyle drugs. This drug has transformed the lifestyles of millions and greatly increased the revenue of many pharmaceutical companies. With the Indian economy growing rapidly at an annual rate of 8-9%, a new era of drug discovery and development coupled with an enormous increase in the marketing of new drugs is being seen. This has certainly made the Indian public vulnerable to issues related to lifestyle drugs. There is a need to study this concept deeply and the impact of these drugs on Indian society, particularly since this topic has already been the centre of many discussions in other developed nations.

Alcohol is the oldest of the lifestyle drugs that have been used since ages in all civilizations. While, sildenafil citrate is currently representing a whole new class of drugs, a turning point in the era of modern lifestyle drugs that has altered the lifestyle of millions, transformed the pharmaceutical industry, and added vigour to the economy. The past few years have also witnessed an altogether new India with respect to drugs marketing and development[1]. The Indian economy has been growing at around 8–9% over the last few years[2]. Although a growing economy is a good sign for country’s development but it may also expose certain vulnerabilities such as exorbitant prices of drugs. Even though a faction of population may afford to buy these drugs but what about the lower socio-economy class? The change in lifestyle particularly in the urban society gave rise to the marketing of lifestyle drugs. There is a need to study the concept of the lifestyle drugs and its impact on society particularly in India as the topic has already been discussed at large in other developed nations[3–5].

## HOW TO DEFINE LIFESTYLE DRUGS?

The term ’lifestyle drug’ is very difficult to define absolutely. Over the last few decades, ‘lifestyle drugs’ and ‘lifestyle medicines’ have been used with increasing frequency, but, no clear-cut definition and demarcation is ever suggested. Møldrup *et al*.,[6] tried to evaluate the quality and quantity of such terms in scientific literature and found no acceptable definition of these terms. The term is also used to describe medicines that are used to treat ‘lifestyle illnesses’, that is to say diseases that arise through ‘lifestyle choices’ such as smoking, alcoholism or overeating, and there are many other shades of meaning as well[7]. In total, 23 different definitions are presented in the scientific literature. Time has come to properly define the term lifestyle drugs and lifestyle medicine.

### Lifestyle drugs should not be interchanged with lifestyle medicine:

‘Lifestyle Medicine’ is an established branch of medicine where we discuss lifestyle’s contribution to health in addition to non-pharmacological intervention in the treatment and management of lifestyle diseases, such as exercise in diabetes mellitus and weight management in obesity[8]. Whereas, in the current scenario, the most operational definition of ‘lifestyle drugs’ is as follows; drugs that could modify or change non-medical or non-health-related goal or conditions at the margins of health and wellbeing[3,7]. These can be used fashionably over the counter to alter not only the appearance but also the physical and mental capabilities (Table 1)[9–13].

**TABLE 1:** LIFESTYLE DRUGS AND THEIR INDICATIONS

Lifestyle drugs	Lifestyle indications
Viagra (Sildenafil citrate)	Impotency, Erectile dysfunction
Norethisterone	Short / postpone menstruation
i-pills (levonorgesterol)	Post-coital contraception
Hormone replacement therapy (HRT)	Improving post-menopausal problems
Anabolic steroids, clenbuterol	Muscle building, physical endurance
Lipase inhibitor (Orlistat), sibutramine	Weight loss
Height increase pills	Height
Benzodiazepines, SSRIs, Marijuana	Mood-alteration, social anxiety disorders
Nicotine Replacement Therapy, bupropion	Cessation of smoking (nicotine)
Caffeine, amphetamine	Memory loss, cognitive enhancers
Minoxidil, finasteride	Baldness
Antioxidants, botulinum toxin	Wrinkles and ageing / cosmetic alterations
Melatonin	Sleep remedy and jet lag remedy
Cyproheptadine	Appetite enhancer
NSAIDs	Work related fatigue
Food supplements, vitamins	General wellbeing

According to the WHO[14], counterfeit medicine is defined as those which have been deliberately and fraudulently mislabeled with respect to identity and/or source. Counterfeiting can apply to both branded and generic products; and counterfeit products may include products with correct ingredients, wrong ingredients, without active ingredients, with insufficient quantity of active ingredient or with fake packaging. This definition as a consequence may includes all types of ‘spurious and fake drugs’, ‘off labels drugs’ including ‘lifestyle drugs’[7]. Thus, as a part of counterfeit medicine strategy, lifestyle drugs should too be tackled as other counterfeit medicines.

### Why are lifestyle drugs booming?:

The lifestyle drugs market is currently worth a phenomenal $29 bn from its starting value[15]. The boom in the growth of lifestyle drugs is suggested to be a complex interaction of vested interests of pharmaceutical industries, surfacing of growing insecurities in the modern day man and the availability of 24×7 telecasting tools of many media. A person in the modern world seeks a very simplistic approach. Everybody tries to solve the problem in a very reductionist, mechanical and biomedical way. They wish to search the answer for every simple health problem in a pill. This bent of the human psyche has further been exploited by some pharmaceutical industry, which obviously has an interest in selling all sorts of pills. These pills may then be dominated by quacks and other media.

Like any business, some drug companies also make decisions about what products to develop based on incentives. They want to sell the most products to the most people as if the money is going to fix the little problems of everyone instead of fixing the big problems of just a few people. It, in fact, generates the demand and then proceeds to meet it. Pharmaceutical companies are actively involved in sponsoring the definition of diseases and promoting them to both prescriber and consumers which is appropriately described by Moynihan *et al*., as disease mongering[16]. “Disease mongering can include, turning ordinary ailments into medical problems, seeing mild symptoms as serious, treating personal problems as medical, seeing risks as diseases, and framing prevalence estimates to maximize potential markets”.

Contemporary medical research is funded on large scales by pharmaceutical companies, some critics of lifestyle drugs worry about medicalization of conditions, meaning that conditions that might be treated with behavioral modification or personal changes move along the continuum toward “medical necessity”, and require health professionals to study, diagnose, prevent and treat. These ailments which were previously considered minor have now come under treatable medical conditions under the influence of various pressures. Latest is an example of medicalization of female dysfunction as a disease and looking for a Viagra-like alternatives for women[17]. Health conditions occur along a continuum. On one end of spectrum lies the situation where treatment offers considerable benefit but there are other situations where these results from treatment are dubious and pose a challenge in certain groups of patients.

Clearly, there is some ambiguity. A school of thought would support lifestyle drugs in the name of progress, indicating that science redefines optimal health constantly, while the other is against them. Evolution of thought is evolving faster in modern age; a common notion that a condition which is occurring outside the domain of a treatment today, may be changed in future. Such evolution of thought is currently occurring generally in conditions with obesity and smoking.

### Lifestyle drugs in Indian perspective:

Two months after the launch of Viagra on December 26th, 2005, Pfizer has exceeded its targets by capturing 1.8% of the market that is estimated to be worth Rs. 80 crore[18]. Is it the tip of iceberg or an indicator of what lifestyle drugs could do to India? Most people unfortunately are inclined to accept a pill as the answer to all life’s problems. Attempts to increase longevity through drugs go back to our earliest record such as using of “*Amrit* (Nectar of Immortality)” that would make an individual to live forever. Several kings wished to remain young and youthful so that they could enjoy the worldly pleasures befitted for their royal existence. Earliest evidences of papyrus during Assyrian and Egyptian civilization witness, the intention of man to live longer[19]. Such a concept of long life has been exploited more so in India by designing drugs and remedies; over the period of time, this scope would further broaden the existence of these lifestyle drugs and its importance by continuous debates.

Because of no public health insurance scheme, 80% of Indian population is spending money out of the pocket on health sector, which is expected to lead poverty by more than 2%[20]. If the same trend including the use of more lifestyle drugs goes on, then chances of increased proportion of population below poverty line will be enlarged. The growth of middle class in the country has resulted in fast changing lifestyles in urban and to some extent rural centers.

As of now, lifestyle drugs are common in the parlance of affluent class but in due course of time, it might spread to other social classes, virtually untapped so far. Similarly, in a scenario where 14% and 4% of health care payments in India respectively are borne by government and insurance sectors, it is pertinent to discuss an issue whether these government and insurance companies should include the payments for lifestyle drugs[21]? In future both these sectors would try to influence our health care payments!

Against the background of these factors, a huge market for lifestyle drugs is in the offing, which hitherto has a very low contribution to the Indian markets. The market is likely to get further intensified, widened and deepened. The growth potential of lifestyle drugs in India is a complex interplay of misplaced priorities of the pharmaceutical industry, rising media especially 24×7 telecasting and robust economic growth in the last few years. The pharmaceutical companies are actively searching for new lifestyle products and conditions.

Since, the lifespan of a human being is increasing and hence the demand for many lifestyle drugs is also burgeoning in a large scale. Keeping this large population in the backdrop, imagine what would happen to India, where there is a young population of 1.2 billion! Fueled by ample and frequent direct to consumer advertising, these lifestyle drugs could have some devastating consequences on young, vibrant and ambitious Indian population. The blatant advertisements and unceasing flaunting of products with supposed improvement in the physical, mental and sexual performances lead to an assault on the fragile mind of youngsters. This is a matter of serious psycho-sociological concern. Mental and emotional health can be corroded by the steady destruction of self-esteem by these kinds of advertisements.

Our drug licensers should not be influenced by the approvals in the West. As most of the medicines are freely available as OTC in India, under such circumstances, there is a need to watch that legitimate lifestyle drugs do not become drug of abuse. Cognition enhancing drugs, stimulants like modafinil, drugs increasing the sexual performance are the potential candidates in this category.

### A case as an instance:

It’s a myth that taller people do better at sports, and height also plays an important role in decisions related to employment, politics and choice of marital partners and *vice versa*[22]. Many companies are thus producing height increase pills worldwide. It is publicized that height increase pills are essential for shorter persons! For the same reasons height increasing pills are widely available in India as OTC products and are widely advertised through advertisements in newspapers and TV channels. These height increase pills or herbal products are assumed to be free from adverse effects! Tall claims are made that these medicines increase height up to 4-5 inches even after your 30th birthday? Such a common availability of dubious drugs lead to many issues; whether these pills are really worth anything? For example, who grows after puberty or 25 years of age? As per the laws of the Drugs and Magic Remedies (Objectionable Advertisements) Act in India, no person can claim to increase height using any medicine; it is punishable under the law. The efficacy of these height increase pills is not known. Probably there might be some loopholes which are being exploited. Indian lawmakers should consider these ineffective “herbal” medicines and start protecting consumers’ interests, as consumers are spending their valuable cash on these nonsense?

### Implications:

Implications associated with labeling of indications and products sales of these lifestyle drugs may be varied. Drugs can, over time, switch from ‘lifestyle’ to ‘mainstream’ use. For example, atropine was first used as a beauty aid based on its ability to dilate the pupil. Cocaine was first described as a lifestyle drug in use by the Indians in South America. It ’satisfies the hungry, gives new strength to the weary and exhausted and makes the unhappy forget their sorrows’, so said Garcilaso de la Vega in 1609. Subsequently assimilated into European medicine as a local anaesthetic, it is now largely returned to lifestyle drug status and, regrettably, is the basis of an illegal multimillion dollar international drugs industry. Cannabis is another good example of a drug that has been considered (in the west at least) as a purely recreational drug but which is now (as tetrahydrocannabinol) in clinical trial for the relief of chronic pain and nausea[7,9,23]. Some of the other implications include, social and cultural, resulting from the use of lifestyle drugs, which is changing the very social fabric of our culture. This increase in their use raises the question of whether we are trying to homogenize the society, consciously or subconsciously! Healthcare systems face an altogether difficult challenge indeed! Should consumers not be protected from ineffective medicines that cost money and distract them from real health problems? Most of the lifestyle drugs are herbal products that are allowed for over-the-counter sales without a prescription. Medical harm resulting from the indiscriminate use of lifestyle drugs includes adverse effects, abuse potentialities and safety concerns. No side-effects claim can also mean that these drugs might have no efficacy either? These easily available ‘lifestyle drugs’ is revolutionizing the traditional relationship of doctor and patient, and raise issues about the rights to, and limits of, self-diagnosis, self-prescription, internet prescription, direct to consumer advertisement (DTCA) and self-medication. Finally the implications to the regulatory system include, evidence-based decision making, efficiency, ethics, laws and standards of regulatory policy to name a few. Another major concern area for the regulatory is the online “free samples” of ‘lifestyles drugs’ such as food supplements, vitamins and drugs like sildanefil, which are directly supplied to consumers.

## CONCLUSIONS

India’s biggest remaining challenges are education and health care, according to the present Finance Minister. In health care, despite some progress, India still has a high rate of maternal and infant mortality, malnourishment, and other endemic rural health problems like cholera, malaria, and other eradicable and communicable diseases. At this point of time we cannot afford to deride India’s growth by misplaced priorities. Industry has a significant contribution in the country’s growth. In order to influence drug industry decisions, India needs to be clear about the drugs policies. The government has an important role in helping us understand what drugs are available, and what drugs we will require in future. In a free market system, profits may be an indicator of what we want as individuals, but they may not be the best indication of what drugs we need as a society.

Drugs that are advertised inappropriately, should be reported to the DCGI/FDA of the state or CDSCO. A project like GPHF minilab has a long way in reducing the negative impact of counterfeit medicines including all sorts of lifestyle drugs. If timely action is not taken then a situation like “Pharmageddon” may be created similar to the movie “Armageddon” (1998), in which the world was saved by a few cowboys; but Pharmageddon would be far more serious. Pharmageddon is defined as “a scenario wherein medicine and medicines produce more ill-health than health, and medical progress does more harm than good”.
